# The emotional geography of National anthems

**DOI:** 10.1038/s41598-025-08956-6

**Published:** 2025-07-02

**Authors:** Petri Toiviainen, Martín Hartmann, Friederike Koehler

**Affiliations:** Department of Music, Art and Culture Studies, Centre of Excellence in Music, Mind, Body and Brain, University of Jyväskylä, PO Box 35, Jyväskylä, 40014 Finland

**Keywords:** National anthems, Music and emotion, Computational music analysis, Geographical patterns, Cultural dimensions, Music information retrieval (MIR), Human behaviour, Computer science

## Abstract

National anthems serve as powerful symbols of national identity, often evoking strong emotional responses. While prior research has examined anthem lyrics, the emotional content of their musical features remains underexplored. This study employs computational modeling to predict the perceived emotional characteristics of 176 national anthems and investigates geographical and cultural variations. Using perceptual data from a prior study and musical features extracted with the MIR Toolbox, we trained LASSO regression models to predict eight emotional characteristics: Valence, Energy Arousal, Tension Arousal, Happiness, Sadness, Tenderness, Anger, and Fear. The predicted emotions were analyzed for continental differences, correlated with latitude and longitude, and compared to Hofstede’s cultural dimensions. The results revealed significant geographic trends, with Valence lower in the Americas and Energy Arousal higher near the equator. Fear and Tension Arousal were more pronounced in the Americas, while Happiness was highest in Oceania. Cultural analyses indicated that hierarchical societies exhibited more energetic anthems, individualistic cultures had less tense but more tender anthems, and indulgent societies expressed greater Fear. These findings highlight the role of musical features in shaping anthem emotions and underscore the potential of computational approaches for large-scale music-emotion research.

**Supplementary Information:**

The online version contains supplementary material available at 10.1038/s41598-025-08956-6.

## Introduction

National anthems represent an important national symbol^1^. Since the formation of nation-states, political leaders have consistently created and adopted national symbols (e.g., flags, anthems, mottos, currencies, constitutions, and holidays) to build and maintain national identity among the country’s population^2^. National anthems serve as official patriotic symbols (often seen as a musical counterpart of the flag), reflecting the nation’s identity and character including its mood, aspirations and goals as defined by leaders^3^. Their main function is to express a nation’s internal unity (establishing bonds and collective goals) and external uniqueness (distinguishing and confirming boundaries)^3^. Government elites thus strive to widely disseminate the national anthem among the population (e.g., incorporating the anthem into school curricula or official events and ceremonies) to legitimate formal authority (also termed banal nationalism^4^). Indeed, due to the emotional potency of music, national anthems are considered powerful tools to shape opinions and behavior across diverse areas, including religion, politics, and war^5^.Music can convey national identity in two ways: from the inside-looking-in (sense of belonging and membership) and outside-looking-in (recognized by non-members)^6^.

### Content and music of National anthems

While the function of national anthems is similar across nations, their content and structure differs greatly among nations, representing different strategies to convey thoughts, emotions, messages, and goals^3^. A broad classification has been consolidated between honor anthems paying (religious) homage and revolutionary anthems^7^. Factors that influence the creation or adoption of an anthem are numerous, including a nation’s form of government, geographic location, socio-political events (e.g., wars, revolutions), economic aspects (e.g., world-system position, modernization) or the creative style during the anthem’s creation or adoption^1^. Most of previous research on national anthems, however, focused on sociological and linguistic analyses. For instance, a cross-cultural lyrics analysis identified diverse topics in national anthems, including ancestry and past, homeland, beauty, unity, victory, or freedom, showing weak correlations with societal features (e.g., age of country, gross domestic product)^8^. Further research found that the lyrical sentiment of national anthems varies by region: Latin and Mediterranean anthems are generally neutral, while Central and Western Asian, Germanic, and Slavic anthems have a more positive sentiment^9^. One content analysis even linked positive messages in anthems to lower suicide rates, while negative or conflicted themes were related to higher suicide rates^10^. However, a critical stance and controversies have emerged during the past decades regarding the use and content of national anthems promoting patriotism, propagandism and chauvinism^11^, especially in music education.

Apart from the lyrical content, the musical structure of national anthems has mainly been investigated in the fields of historical musicology and ethnomusicology through case studies, for instance, on the anthem of Zimbabwe^12^. Earlier sociological work has linked symbolic musical codes in anthems with sociopolitical control and a nation’s world-system position at the time of the anthem’s creation or adoption^1,3^. However, a systematic and comprehensive investigation of objective musical features in a variety of anthems has received little academic attention yet, especially regarding the emotional content of the music. An earlier investigation of anthems of 18 European countries^13^ revealed a positive association of the proportion of low notes in national anthems with students’ perceptions of the anthems’ gloominess and sadness as well as with national suicide rates. A recent initial study based on computational music analysis^14^ points to possible links between certain musical characteristics (e.g., low pitch, high tempo, high beat) and positive social outcomes (e.g., high happiness and peace, low suicide rate). Although the main function of music lies in its potency to induce and affect emotions^15^, little is known about the emotions reflected in national anthems and potential underlying influencing factors, such as geographical location or cultural differences.

### Music and emotions

The emotional characteristics of national anthems might be explained by several underlying factors. These include cultural movements, national tendencies and environmental factors such as geographic and climatic differences between countries. Before digging deeper into this issue—and particularly on the role of geography and cultural orientation upon emotions expressed by anthems, which is a central topic to this study—, it is necessary to introduce the main psychological and computational frameworks used in music and emotion research.

Emotions in music can be experienced as a subjective response to music (i.e., felt or induced emotions) or be attributed to music (i.e., expressed or perceived emotions), although there might be a significant overlap in this classification^16^. The most prominent theoretical frameworks used in music and emotion research are discrete and dimensional models of emotions^17,18^. According to Ekman’s discrete or basic emotion model^19^, all emotions can be traced back to a small set of fundamental and inherent emotions (fear, anger, disgust, sadness, and happiness), with specific underlying neurophysiological systems. The two-dimensional circumplex model^20^, however, proposes that all emotions emerge from two independent fundamental dimensions, that is, valence (a pleasure–displeasure continuum) and arousal (activation–deactivation). Later work suggested an expansion into a three-dimensional model through dividing arousal into two separate dimensions: tension arousal and energy arousal^21^. Both main classes of models have been commonly applied to investigate emotions in music, while it has also been argued that it may not capture the complexity of emotions in an aesthetic context like music^22^. For instance, the basic emotion of disgust has often been modified to tenderness in the context of music research^18^. One of the main frameworks to explain music-evoked emotions (BRECVEMA)^23^ suggests seven underlying mechanisms (brain stem reflex, rhythmic entrainment, evaluative conditioning, contagion, visual imagery, episodic memory, musical expectancy, and aesthetic judgment), with brain stem reflex, rhythmic entrainment, and musical expectancy being mostly dependent on the musical content.

Apart from measuring emotions associated with music through directly asking individuals (self-reports), recent innovative approaches have included music emotion recognition (MER), that is, the computational task of automatically recognizing emotional content in music or emotions induced by music. MER is a high-level problem within the field of music information retrieval (MIR), an interdisciplinary area focusing on understanding and organizing music collections using computational techniques. MER has several applications, such as automatically categorizing music pieces based on emotions or recommending music tailored to a user’s mood^24^. MER systems can be built upon musical content and/or context and include user factors such as demographic or situational information^25^. In a typical MER framework, signal processing techniques are used to extract emotionally relevant features from music excerpts. Audio-based features representing musical dimensions such as loudness, timbre, rhythm and harmony have been extensively studied in MER^26^. These features are paired with ground truth data—human annotations that describe perceived or induced emotions. A machine learning model is then trained on part of this annotated data set to recognize patterns, and its performance is evaluated on the remaining data. While previous research on national anthems has predominantly focused on the lyrical content and less on the emotional content as reflected in the music itself, MER offers great potential for an objective and comprehensive analysis of the emotions expressed in national anthems, elucidating certain patterns and providing insights into cross-cultural comparisons.

### Emotions in anthems: geography and cultural differences

Some of the similarities and differences in the emotional characteristics of national anthems might be explained through identifying geographical patterns. Geographical location often shapes the cultural, historical, and social context of a nation and the development of its identity. For instance, the history of a broader region, including events like wars, revolutions, and independence movements, often occurs in reciprocity with nearby countries and might impact the content and emotional tone of national anthems. Understanding the geographical context might help to interpret these historical influences and how they are reflected in the music. Furthermore, the location of a country and its surrounding landscape and resources are prominent themes in national anthems^8^, potentially influencing the music as well. As a recent example, Saudi Arabia, the largest petroleum exporter, is reportedly rearranging its national anthem with a Western composer, potentially incorporating new influences that reflect both its identity as a resource-rich nation and its increasing Westernization amid economic diversification efforts^27,28^.

Variations in national anthems might also be linked with differences in cultural values and behaviors. A widely used framework to understand cultural differences (Hofstede’s cultural dimensions theory)^29,30^ proposes six main cultural dimensions: *Power Distance* (PDI; solutions to the basic issue of human inequality), *Individualism vs. Collectivism* (IDV; integration of individuals in primary group), *Motivation towards Achievement and Success*, formerly *Masculinity vs. Femininity* (MAS; preference for achievement or cooperation), *Uncertainty Avoidance* (UAI; stress facing an unknown future), *Long-Term vs. Short-Term Orientation* (LTO; focus on future, present, or past), and *Indulgence vs. Restraint* (IVR; regulation of human desires of enjoyment). These dimensions have been consistently used to examine important differences among nations in terms of political and economic systems, business and management practices, and other societal variations^31^. However, to the best of our knowledge, no study has investigated these dimensions regarding differences in national anthems yet.

### Research objectives

Based on the lack of research on the emotions reflected in the music of national anthems and their possible links with geographical location and cultural differences, the main research objective of the study is to provide an overview of the emotional geography of national anthems based on computational modeling. Specifically, it aims to:

- analyze the emotional content of national anthems based on their musical features using computational modeling.
- examine geographical patterns in the emotional characteristics of national anthems, focusing on both continents and specific coordinates (latitude and longitude).
- compare the predictability of emotion dimensions (Valence, Energy Arousal, Tension Arousal) and basic emotions (Happiness, Sadness, Tenderness, Anger, Fear) from musical features.
- explore how emotional expressions in national anthems vary globally and reflect broader cultural differences (Hofstede’s cultural dimensions).

## Methods

We used computational modeling because it allowed us to concentrate on how the contribution of specific musical elements—such as timbral, rhythmic, and tonal characteristics—to emotional expression varies globally without interference from factors like lyrical content, cultural context, patriotic sentiment, or political associations. This approach enabled us to focus on structural musical features that are comparable across countries and to generate systematic emotion estimates that reflect only the acoustic and musical content of the anthems themselves.

### Material

The material used in the study comprised instrumental recordings of national anthems, sourced from the comprehensive database *National Anthems.info*^32^. A deliberate choice was made to include only those anthems presented in authentic instrumental form, thereby excluding any renditions played using MIDI instruments to ensure the acoustic consistency and authenticity of the sample. As a result, a total of 176 anthems were selected for further analysis. This selection process involved choosing the most recent anthem for countries with a history of multiple anthems, aligning with our focus on contemporary national identity as reflected in these musical symbols. The final dataset exhibits a global representation, with anthems from Europe (43), Asia (40), Africa (50), the Americas (35), and Oceania (8), ensuring a broad cultural and geographical scope for the analysis. The average length of the anthem recordings was 83.7 s, ranging from 31.8 s (Estonia) to 270.0 s (Uruguay). The list of countries included in the study is detailed in Supplementary Table 1.

### Emotion modelling

As we were interested in how the contribution of specific musical elements—such as timbral, rhythmic, and tonal characteristics—to emotional expression varies globally, computational modeling allowed us to focus on these features without interference from factors like lyrical content, cultural context, patriotic sentiment, or political associations. To this end, we combined perceptual data from the emotion database of Eerola^33^, which includes musical excerpts with diverse emotional content, with musical features extracted by music information retrieval techniques. We trained eight LASSO models using the Soundtracks dataset and applied them to predict the emotional content of the national anthems. To ensure the models’ generalizability, we applied cross-validation and regularization, minimizing overfitting and enhancing their applicability to unseen data. Finally, we used the resulting models to predict the perceived emotional content of the national anthems, linking musical characteristics with emotional perception in a data-driven and systematic manner. It must be noted that the emotion ratings in the Eerola dataset were primarily collected from Finnish participants, the resulting models may reflect culturally specific perceptual norms. This potential bias may limit their generalizability across cultural contexts and should be considered when interpreting the predicted emotional values.

#### Musical feature extraction

The modelling of emotional content was grounded in the analysis of 360 film soundtrack excerpts, using the emotion database of Eerola^33^. This database comprises ratings of eight emotion characteristics provided by 116 participants. The emotion characteristics were based on two models: a dimensional model encompassing valence, energy arousal, and tension arousal, and a basic emotions model including happiness, sadness, tenderness, anger, and fear. Utilizing the MIR Toolbox^34^, we extracted 65 musical features, representing dynamic, rhythmic, spectral, timbral, and tonal content, from each movie soundtrack excerpt. The extracted features are shown in Supplementary Fig. 1. The analysis adopted a bag of frames approach, calculating both means and standard deviations of the features, thus capturing the dynamic range and variability within each excerpt. Features with a skewed distribution were Box-Cox transformed. As the result of the feature extraction stage, each of the soundtrack excerpts was represented by a 65-dimensional feature vector.

#### LASSO regression

To link the extracted musical features to the emotion ratings, we employed least absolute shrinkage and selection operator (LASSO) regression^35^. This method was chosen for its effectiveness in handling multicollinearity and selecting relevant predictors in datasets with many variables. LASSO, or Least Absolute Shrinkage and Selection Operator, is an iterative regression technique that imposes a penalty on the absolute size of regression coefficients, effectively reducing less relevant predictors to zero and retaining only the most influential ones. This approach enhances the generalizability of the model by focusing on a parsimonious set of predictors that are robust across different datasets.

We treated each emotion characteristic as a dependent variable in separate models and assessed the predictive power of the musical features through cross-validation, using an 80/20 random split into training and testing sets across 100 runs to ensure robust findings. We determined the optimal regularization parameter by minimizing the prediction error for the test data, based on the average model accuracy measured by the adjusted coefficient of determination across these runs. This parameter controls the degree of shrinkage applied to the regression coefficients, balancing model complexity and generalizability. Finally, we retrained the models using the full dataset and the identified optimal regularization parameters to enhance predictive accuracy.

Table 1 shows the optimal regularization parameters and model accuracies (adjusted coefficients of determination) for each emotion characteristic, with accuracies reflecting average performance on held-out test sets across the 100 cross-validated training runs. As shown, the three emotion dimensions—Valence, energy arousal, and tension Arousal—are predicted with greater accuracy than the basic emotions. This difference is expected, as emotion dimensions represent broader, more generalized affective constructs, while basic emotions are more discrete and context-dependent, making them harder to model. Furthermore, tenderness exhibits the lowest optimal regularization parameter, indicating reliance on a broader set of features, whereas happiness has the highest, suggesting a more constrained set of contributing predictors.

**Table 1:** Optimal regularization parameters λ_opt_ and corresponding model accuracies (adjusted coefficients of determination) for each emotion characteristic. Accuracies reflect average performance on held-out test sets across 100 cross-validated training runs.

Emotion	λ_opt_	Adjusted *r*^2^
Valence	0.0480	0.6728
Energy arousal	0.0316	0.7312
Tension arousal	0.0358	0.6913
Anger	0.0551	0.6112
Fear	0.0369	0.6487
Happiness	0.0897	0.4783
Sadness	0.0601	0.5555
Tenderness	0.0261	0.6148

### Emotion prediction

After training the eight LASSO models, we extracted the 65 musical features illustrated in Supplementary Fig. 1 for all 176 national anthems. Each anthem was thus represented by a 65-dimensional feature vector. Using the trained models, we predicted the eight perceived emotion characteristics for each anthem.

### Emotions of anthems and geographical variation

Using the predicted emotional characteristics, we subsequently analyzed their regional differences through a comparison between individual countries as well as across continents using one-way ANOVAs. We further examined global patterns by performing correlation analyses between the predicted emotional characteristics and geographical coordinates, including latitude and longitude. The geographical centroids of countries were sourced from the *Climate Data Toolbox*^36^.

It is important to recognize the distinct characteristics of latitude and longitude as geographical variables. Latitude is linear and directly linked to the Earth’s geography, representing the distance north or south from the equator. Accordingly, we used latitude values, $\:\phi\:$, as well as their absolute values, $\:\left|\phi\:\right|$, directly in the correlation analysis. Longitude, by contrast, is cyclical, requiring periodic wrapping to ensure continuity. Additionally, the origin of longitude at the Greenwich Meridian is a cultural convention established during the International Meridian Conference of 1884, rather than a natural reference point. Consequently, for longitude ($\:\lambda\:$), we set its values to increase from west to east, applied a sine transformation, calculated as $\:sin(\lambda\:-\delta\:)$, and determined the value of the longitude offset, $\:\stackrel{\sim}{\delta\:}$, that maximized the absolute value of correlation. This transformation accounts for the cyclical nature of longitude and allows for directional correlations that are invariant to arbitrary zero points such as the Greenwich Meridian.

### Emotions of anthems and cultural dimensions

To explore the influence of societal values and behaviours on the emotional content of national anthems, we conducted correlation analyses between the predicted emotional characteristics and Hofstede’s^29,30^ six cultural dimensions. The availability of data varied across the six scales: PDI, IDV, MAS, and UAI included data for 66 countries, while LTO and IVR included data for 89 countries. The countries associated with each scale are listed in Supplementary Table 1.

## Results

The prediction accuracies of the models used to describe the emotion characteristics of each anthem were moderate to moderately high, as shown in Supplementary Table 2. This table presents the regularization parameter and coefficient of determination for the optimal LASSO model corresponding to each emotion characteristic. Emotion dimensions (Valence, Energy Arousal, and Tension Arousal) were predicted with greater accuracy than basic emotions due to their broader and more generalized representation of emotional content. The model coefficients are illustrated in Supplementary Fig. 1.

### Emotions of anthems per country and continent

#### Emotion dimensions

**Fig. 1:**
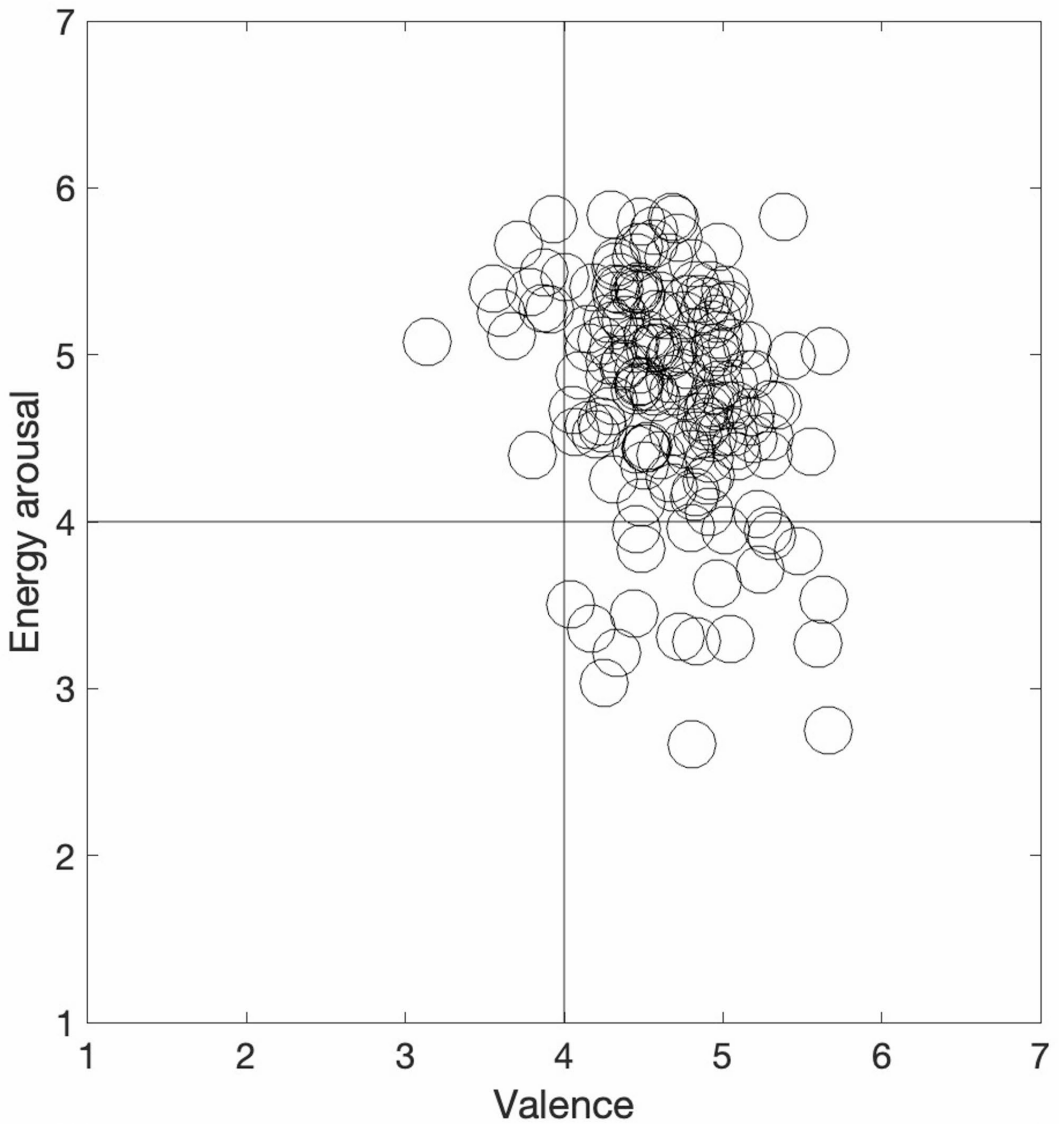
Scatter plot of Valence and Energy Arousal of the anthems.

Figure 1 displays a scatter plot of the Valence and Energy Arousal of the 176 national anthems, as predicted by the respective LASSO models.

As can be seen, most of the anthems are located in the quadrant characterized by positive Valence and high Energy Arousal, indicating that happiness is the predominant basic emotion they convey.

**Fig. 2:**
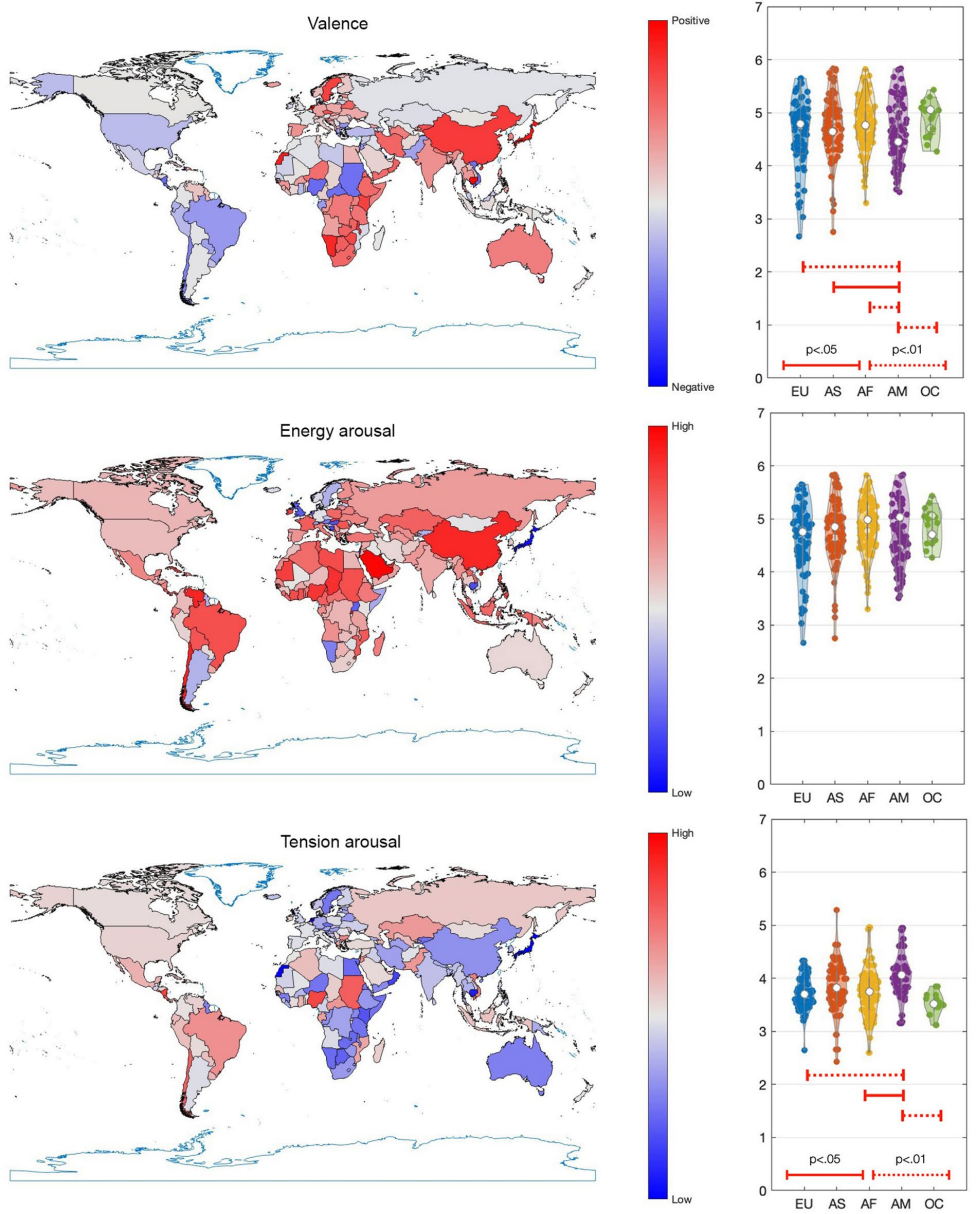
(Left) Emotional content of each country’s anthem for Valence, Energy Arousal, and Tension Arousal. For each emotion, the values have been linearly scaled such that the minimum value is displayed in blue and the maximum value in red. (Right) Violin plots of emotion dimensions per continent. EU = Europe, AS = Asia, AF = Africa, AM = Americas, OC = Oceania. Significant differences obtained from pairwise t tests (FDR corrected) are indicated with solid and dotted line segments for adjusted p thresholds of 0.05 and 0.01, respectively.

Figure 2 (left side) presents Valence, Energy Arousal, and Tension Arousal of anthems displayed on a world map. The values are scaled linearly such that the minimum value is displayed in blue and the maximum value in red.

While there is a great extent of local variation in the emotional content, some geographical trends can be observed. For instance, Valence tends to be more negative in the Americas compared to other regions. Energy Arousal appears to be higher in countries situated close to the equator, while many countries in Southern Africa and South Asia, as well as Australia exhibit lower Tension Arousal, indicating a calmer emotional tone in the anthems from these regions. Table 2 provides an overview of the countries with the three lowest and three highest values for each emotion dimension.

**Table 2:** Anthems with lowest and highest values of each emotion dimension.

Emotion dimension	Lowest	Highest
Valence	Qatar, US Virgin Islands, Sudan	Japan, Western Sahara, Netherlands
Energy Arousal	Luxemburg, Japan, Liechtenstein	Kuwait, Saint Kitts and Nevis, China
Tension Arousal	Japan, Western Sahara, Netherlands	Qatar, Nigeria, US Virgin Islands

Subsequently, we conducted a series of one-way ANOVAs to examine differences in Valence, Energy Arousal, and Tension Arousal of anthems across continents. The results revealed significant continental differences for all three dimensions. Valence exhibited a significant effect of continent (*F*(4, 170) = 5.87, *p* <.001, η²=0.12), with means ranging from 4.39 to 5.00. Energy Arousal also showed significant differences (*F*(4, 170) = 2.48, *p* =.046, η²=0.055), with means between 4.54 and 4.93. Tension Arousal demonstrated a strong continental effect as well (*F*(4, 170) = 4.65, *p* =.001, η²=0.097), with means varying from 3.51 to 4.05.

Next, we conducted post hoc tests using pairwise *t*-tests with false discovery rate (FDR) correction to account for multiple comparisons. Figure 2 (right side) presents violin plots for each of the emotion dimensions, illustrating the distribution of scores, and displays pairwise significant differences identified through FDR-adjusted *q* values (< 0.05). Most notably, Valence in the Americas was significantly more negative than in all other continents, reflecting a distinct emotional tone in anthems from this region. Additionally, Tension Arousal in the Americas was higher compared to Europe, Africa, and Oceania, suggesting that anthems from the Americas convey a heightened sense of urgency or intensity relative to those from these other continents.

#### Basic emotions

**Fig. 3:**
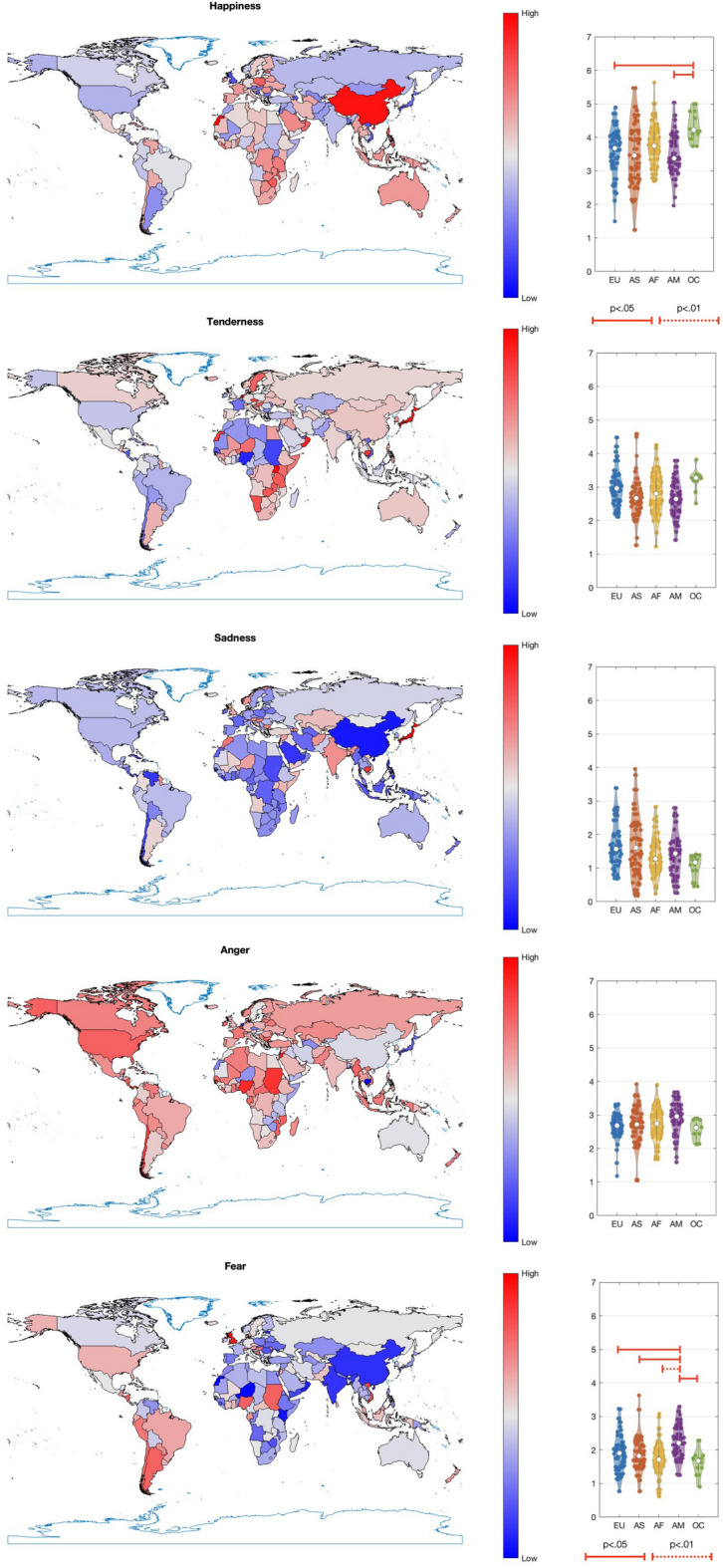
(Left) Strength of Happiness, Tenderness, Sadness, Anger, and Fear of anthems per country. For each emotion, the values have been linearly scaled such that the minimum value is displayed in blue and the maximum value in red to cover the entire range of colours. (Right) Violin plots of basic emotions per continent. EU = Europe, AS = Asia, AF = Africa, AM = Americas, OC = Oceania. Significant differences obtained from pairwise t tests (FDR corrected) are indicated with solid and dotted line segments for adjusted p thresholds of 0.05 and 0.01, respectively.

Figure 3 displays the emotional content in terms of the five basic emotions per country. The values are scaled linearly such that the minimum value is displayed in blue and the maximum value in red.

Similar to the emotion dimensions, there is a significant degree of local variation. We encourage the reader to explore the general trends for each emotion independently.

Meanwhile, Table 3 provides an overview of the countries with the three lowest and three highest intensities for each emotion.

**Table 3:** Anthems with lowest and highest strength of each basic emotion.

Basic emotion	Lowest	Highest
Happiness	Israel, Liechtenstein, Jamaica	Western Sahara, China, Dominica
Tenderness	Nigeria, Qatar, US Virgin Islands	Japan, Cambodia, Netherlands
Sadness	Lebanon, China, Rwanda	Japan, Israel, Liechtenstein
Anger	Cambodia, Japan, Netherlands	Qatar, Sudan, Nigeria
Fear	Niger, Kenya, China	Qatar, Jamaica, Liechtenstein

For the basic emotions, the one-way ANOVAs revealed significant differences across continents for Fear (*F*(4, 170) = 5.23, *p* <.001,η²=0.11), Happiness (*F*(4, 170) = 3.31, *p* =.012, η²=0.070), Sadness (*F*(4, 170) = 2.47, *p* =.047, η²=0.055), and Tenderness (*F*(4, 170) = 3.01, *p* =.020, η²=0.066), while no significant differences were found for Anger (*F*(4, 170) = 1.36, *p* =.250, η²=0.031). Again, post hoc tests were conducted using pairwise *t*-tests with false discovery rate (FDR) correction to account for multiple comparisons. Figure 3 (right side) presents violin plots for each of the five basic emotions, illustrating the distribution of scores and highlighting pairwise significant differences identified through FDR-adjusted p values (< 0.05). It can be observed that Fear was significantly higher in the Americas than in all other continents, while Happiness was significantly higher in Oceania than in the Americas and Europe.

### Emotions of anthems and location on the Globe

To gain a more detailed understanding of the relationship between the emotional content of anthems and geographical location, we conducted correlation analyses between the predicted perceived emotions of the anthems and the latitudes and longitudes of the geographical centroids of the respective countries.

#### Emotion dimensions

The correlations between the emotion dimensions and the geographical coordinates of the countries, along with the optimal longitude offsets, are displayed in Table 4.

**Table 4:** Correlation coefficients and their 95% confidence intervals between the intensity of the three emotion dimensions in the anthems and latitude ($\:{r}_{\phi\:}$), absolute value of latitude ($\:{r}_{\left|\phi\:\right|}$), and sine-transformed longitude ($\:{r}_{sin(\lambda\:-\stackrel{\sim}{\delta\:})}$) of the countries’ geographical centroids, as well as the optimal longitude offset ($\:\stackrel{\sim}{\delta\:}$). **p <.01, ***p <.001, two-tailed.

Emotion dimension	$\:{r}_{\phi\:}$	$\:{r}_{\left|\phi\:\right|}$	$\:{r}_{sin(\lambda\:-\stackrel{\sim}{\delta\:})}$	$\:\stackrel{\sim}{\delta\:}$
Valence	−0.012 [−0.160, 0.136]	0.051 [−0.098, 0.197]	0.252*** [0.108, 0.385]	−7°
Energy arousal	−0.142 [−0.284, 0.006]	−0.217** [−0.354, −0.071]	−0.060 [−0.206, 0.089]	0°
Tension arousal	−0.020 [−0.167, 0.128]	−0.075 [−0.220, 0.074]	−0.208** [−0.345, −0.062]	1°

For Valence, the significant positive correlation with sine-transformed longitude (*r* =.252, *p* <.001, 95% CI [0.108, 0.386]) and an optimal longitude offset of −7° suggests that Valence tends to increase when moving towards the east from the longitude 7°W, and vice versa. Energy Arousal, with a significant negative correlation to absolute latitude (*r* = −.217, *p* <.01, 95% CI [−0.354, −0.071]), indicates that anthems from countries closer to the equator exhibit higher Energy Arousal. Finally, Tension Arousal, which correlates negatively with sine-transformed longitude (*r* = −.208, *p* <.01, 95% CI [−0.345, −0.062]) at an optimal offset of 1°, implies a regional increase in Tension Arousal when moving towards the west from the longitude 1°. It is notable that the optimal longitude offset values are close to zero for all three emotion dimensions.

#### Basic emotions

Table 5 shows the correlations between the strength of basic emotions and the geographical coordinates of the countries.

**Table 5:** Correlation coefficients and their 95% confidence intervals between the strength of basic emotions in the anthems and latitude ($\:{r}_{\phi\:}$), absolute value of latitude ($\:{r}_{\left|\phi\:\right|}$), and sine-transformed longitude ($\:{r}_{sin(\lambda\:-\stackrel{\sim}{\delta\:})}$) of the countries’ geographical centroids, as well as the optimal longitude offset ($\:\stackrel{\sim}{\delta\:}$). *p <.05, **p <.01, ***p <.001, two-tailed.

Basic emotion	$\:{r}_{\phi\:}$	$\:{r}_{\left|\phi\:\right|}$	$\:{r}_{sin(\lambda\:-\stackrel{\sim}{\delta\:})}$	$\:\stackrel{\sim}{\delta\:}$
Happiness	−0.219** [−0.355 −0.073]	−0.178* [−0.317 −0.031]	0.065 [−0.183, 0.211]	−79°
Tenderness	0.027 [−0.121 0.174]	0.114 [−0.0342 0.258]	0.099 [−0.050 0.243]	−32°
Sadness	0.249*** [0.105 0.383]	0.247*** [0.103 0.381]	0.134* [0.014, 0.276]	33°
Anger	−0.011 [−0.158 0.137]	−0.090 [−0.234 0.059]	−0.155* [−0.296, −0.007]	−11°
Fear	0.017 [−0.131 0.165]	0.034 [−0.114 0.181]	−0.242*** [−0.377, −0.098]	4°

As can be seen, Happiness shows a significant negative correlation with latitude (r_φ_ = − 0.219, *p* <.01, 95% CI [−0.355 −0.073]) and absolute latitude (r_|φ|_ = − 0.178, *p* <.05, 95% CI [−0.317 −0.031]), suggesting that Happiness is stronger closer to the equator and in the south. Sadness, on the other hand, exhibits significant positive correlations with both latitude (r_φ_ = 0.249, *p* <.001, 95% CI [0.105 0.383]) and absolute latitude (r_|φ|_ = 0.247, *p* <.001, 95% CI [0.103 0.381]), indicating that higher levels of Sadness are associated with regions farther from the equator and in the north. Additionally, Sadness has a weaker, but significant, positive correlation with sine-transformed longitude ($\:{r}_{sin(\lambda\:-\stackrel{\sim}{\delta\:})}$ = 0.134, *p* <.05, 95% CI [0.014, 0.276]) with an optimal offset of 33°E, indicating that Sadness tends to increase slightly when moving eastward from this longitude. Both Fear ($\:{r}_{sin(\lambda\:-\stackrel{\sim}{\delta\:})}$= − 0.242, *p* <.001, 95% CI [−0.377, −0.098]) and Anger ($\:{r}_{sin(\lambda\:-\stackrel{\sim}{\delta\:})}$ = − 0.155, *p* <.05, 95% CI [−0.296, −0.007]) show significant negative correlations with sine-transformed longitude, with optimal offsets $\:\stackrel{\sim}{\delta\:}\:$close to 0°, implying that these emotional qualities tend to become stronger when moving towards west from the zero meridian. By contrast, Tenderness does not exhibit significant correlations with any geographical coordinate, suggesting a lack of strong geographical patterns for this emotion. These findings indicate that Sadness and Happiness are primarily associated with latitude, while Fear and Anger display notable longitudinal patterns.

### Anthem emotions and cultural dimensions

Power Distance (PDI) showed a moderate positive correlation with Energy Arousal (*r*(64) = 0.516, *p* <.001, 95% CI [0.309 0.676]), indicating that countries with hierarchical structures, where unequal power distribution is more accepted, tend to have more energetic anthems. Individualism vs. Collectivism (IDV) exhibited negative correlations with both Energy Arousal (*r*(64) = − 0.373, *p* <.05, 95% CI [−0.567 −0.140]) and Tension Arousal (*r*(64) = − 0.369, *p* <.05, 95% CI [−0.564 −0.136]), suggesting that individualistic societies have less energetic and less tense anthems. Additionally, IDV correlated positively with Tenderness (*r*(64) = 0.383, *p* <.05, 95% CI [0.151 0.575]), implying that these societies favor anthems with gentler emotional tones. Finally, Indulgence vs. Restraint (IVR) correlated positively with Fear (*r*(87) = 0.374, *p* <.01, 95% CI [0.177 0.542]), indicating that indulgent societies, which emphasize enjoyment and gratification of desires, tend to have anthems expressing higher levels of fear.

## Discussion

In this study, we explored the possible connections between the emotional content of instrumental renditions of 176 national anthems and their geographical location. To achieve this goal, we extracted a set of acoustic features describing expressed emotions by the anthems and three geodetic features based on latitude and longitude of the geometric center of the countries. We anticipated geographical patterns, based on the assumption that climatic and biogeographic factors shape the emotional content of national anthems—just as they do with other national symbols like flags, coats of arms, and birds, as well as national identity components such as language, history, ancestry, culture, and cuisine.

### Summary of results

Most national anthems across the studied countries conveyed some degree of Happiness (Fig. 1). However, a cross-continental comparison revealed notable differences in emotional expression (Figs. 2 and 3). Valence was significantly more negative in the Americas than in other continents. Tension Arousal was also higher in the Americas compared to Europe, Africa, and Oceania. Additionally, Fear levels were significantly higher in the Americas than in all other regions, while Happiness was more pronounced in Oceania than in the Americas and Europe.

Latitudinal trends revealed that Energy Arousal was stronger in countries closer to the equator. Happiness was also more prominent near the equator and in the southern regions, while Sadness was more prevalent farther from the equator and in the north. Longitudinally, Valence increased eastward, whereas Tension Arousal and Anger intensified westward. Similarly, Sadness rose in an eastward direction, whereas Fear increased toward the west.

When examining emotions in relation to Hofstede’s cultural dimensions^29,30^, countries with hierarchical power structures tended to have more energetic anthems. In contrast, individualistic societies favored anthems that were less tense, less energetic, and more tender. Additionally, indulgent societies tended to have anthems that expressed higher levels of Fear.

### General discussion

One of the clearest results of this study was that anthems of American countries exhibit higher levels of Fear, greater Tension Arousal, and more negative Valence compared to those from other regions. Analyzing countries with extreme values for key predictors of these emotional features further supports this pattern. For instance, Chile’s and Nicaragua’s anthems feature frequent harmonic changes, Jamaica’s and Aruba’s have low pulse clarity, and Antigua and Barbuda’s anthem has high spectral energy in center frequencies, all of which are strong predictors of Fear. Anthems from Barbados and Dominica are very high in roughness, with their instrumentation dominated by brass and percussive elements, resulting in high Tension Arousal and low Valence. Brazil and Costa Rica exhibit very low key clarity—an indicator of how clearly a piece establishes its tonal center—which is associated with elevated levels of Fear and Tension Arousal.

While these musical features highlight the emotional qualities of anthems, their interpretation can be informed by the broader historical and cultural context. The native music of many of these nations is characterized by pentatonic scales, as seen in Andean cultures^37^, yet postcolonial anthems are heavily influenced by—or even composed by—European musicians of the 19th century. The rich rhythmic and tonal palette of Italian opera and other Romantic music might partly explain the emotional qualities of these anthems. At the same time, these musical choices reflect a decolonization process largely led by elites of mixed European and non-European ancestry, rather than by the broader colonized population^38,39^. In line with this interpretation, another study suggested that the language of an anthem—specifically Spanish—is a significant factor contributing to the bellicose nature of anthems^40^, which may also explain some of the heightened emotional intensity observed.

Another key finding was that anthems from Oceania expressed higher levels of Happiness compared to those from the Americas and Europe. An examination based on mode, a key predictor of Happiness, shows that Fiji’s anthem scored the highest in exhibiting characteristics associated with the major mode compared to all other anthems analyzed. It has also been observed that most Oceanic anthems are uplifting church hymns or patriotic songs from their current or former colonizing countries^32^, whereas European and American anthems are more diverse in that they can be persistently major (e.g., Germany, Bolivia) or have passages in minor mode (e.g., Argentina, France). However, this finding should be interpreted with caution due to the small sample size (8 countries) from Oceania.

Regarding latitudinal trends, we found that anthems from countries closer to the equator exhibit higher levels of Happiness and Energy Arousal, along with lower levels of Sadness. Similarly, countries in southern regions tend to have higher Happiness and lower Sadness. The similarities in correlations between these emotions and both latitude and absolute latitude may stem from the uneven distribution of countries between the Northern and Southern Hemispheres. Examples of this relationship include, Niger, Libya, and Western Sahara, which display the highest pulse clarity in the dataset—a positive predictor of Happiness and Energy Arousal and a negative predictor of Sadness. Additionally, Zimbabwe’s anthem scores exceptionally high in major mode, while New Zealand’s high spectral irregularity is associated with Happiness. Meanwhile, Brazil and Chile’s frequent harmonic changes correspond to lower Sadness. Some of these regions, particularly those near the tropics, may benefit from more pleasant climates that enhance mood, encourage outdoor activities, and promote a more active, social lifestyle^41^.

We found an eastward increase in Valence and a decrease in Tension Arousal and Anger. This pattern is exemplified by the Japanese anthem, which, with its longer notes and minimal percussion, shows decreased spectral roughness (indicating high Valence and low Tension Arousal) and low spectral flux (linked to low Anger). Like Vanuatu’s anthem, it also has very high key clarity (corresponding to high Valence and low Tension Arousal). Compared to the 19th-century Latin American epic anthems, more Eastern anthems^42^ (from Europe, Asia, Africa, and Oceania) are more varied in age and are often better characterized as odes, marches, and fanfares, with higher key clarity and more consistent tempo. However, interpreting these results is challenging and requires further investigation.

Our results show an eastward increase in Sadness. For instance, Eastern Hemisphere countries such as Japan and Cambodia head the list of countries having the lowest harmonic change, and low values on this feature are predictive of elevated Sadness. Contributing to this result might be the use of anhemitonic musical scales in some East Asian anthems, such as the pentatonic scale in China and the *yo* scale in Japan, reflecting the traditional music of these countries^43^. The lack of half steps results in a more uncertain mode than the clearly major mode of other anthems. Also, Japan’s anthem ranks amongst the lowest in rhythmic clarity, a feature that contributes negatively to Sadness. It should be noted that these results have the lowest magnitude and do not align with other findings. Moreover, the distribution of predicted Sadness scores is skewed, with most countries showing low values and a few, such as Japan, exerting disproportionate influence, which may limit the generalisability of the eastward trend.

Hofstede’s cultural dimensions can help further explain the connection between the emotions expressed in anthems and geographic coordinates. Specifically, countries with high Power Distance and collectivist tendencies tend to have more energetic anthems, reflecting a cultural emphasis on collective identity and hierarchical social structures. For instance, Ecuador and Panama—highly collectivistic and power-distant countries—feature anthems with clear rhythmic periodicity, frequent percussion, and high spectral entropy, flux, and brightness. These elements contribute to a more dynamic and forceful anthem, potentially reinforcing collective identity through energetic symbolism. Conversely, countries such as Denmark and New Zealand, which score high in Individualism and low in Power Distance, tend to have more subdued anthems. Their anthems are cantabile, with longer notes and less frequent percussion, creating a less energetic and tense atmosphere. These anthems could be seen as a reflection of individualistic values and egalitarian social structures, where personal autonomy is emphasized and hierarchical power distribution is minimized. It is worth noting that Power Distance is moderately negatively correlated with Individualism (*r* = -.60, *p* <.001), linking to the concept of ‘vertical collectivism’^44^, a cultural orientation combining collectivist values with hierarchical structures and respect for authority.

Another relevant cultural dimension was Indulgence, which significantly correlated with the expression of Fear in national anthems. More broadly, Indulgence is negatively linked with Long-Term Orientation (r(87)=−0.45, *p* <.001, 95% CI [−0.267 −0.602]) and with the Human Development Index, a measure of country development based on health, education and income^45^, (r(87) = −0.31, *p* <.001, 95% CI [−0.487 −0.109]), suggesting that countries with higher indulgence scores tend to experience greater political, economic, or social instability^46^. This may help explain why nations such as Venezuela, Mexico, Puerto Rico, and El Salvador—where Indulgence and short-term orientation are high—also emphasize emotional intensity in their anthems. These anthems often feature more harmonic changes—a positive predictor of Fear—and increased chromaticism. In contrast, more stable countries like Sweden, which rank low in Indulgence, tend to have anthems with greater key clarity, which is a negative predictor of Fear.

### Limitations

While the study provides valuable insights into the emotional characteristics of national anthems, several limitations should be considered. First, our results should be interpreted merely as associations without implying any causality and might be confounded by various unconsidered variables. Second, the study operates on the assumption that emotions modeled from musical features are universally perceived, which may overlook cultural differences in emotional interpretation, such as those found about self-perceived well-being^47^ or to the perception of mode in chords and melodies^48^. Third, the emotion modeling relies on ratings derived from a separate film soundtrack dataset, which may not perfectly align with the emotional characteristics of national anthems. Fourth, the emotion models were based on perceptual ratings from 116 participants, likely from a culturally homogeneous background, which may introduce cultural bias and limit the generalizability of the predicted emotions to non-Western or more diverse populations. Fifth, the use of a model trained on film soundtracks to predict emotions in national anthems presents a limitation. As soundtrack music is typically composed in conjunction with visual narratives, emotions like fear or anger may be expressed differently than in ceremonial or symbolic music. This discrepancy can result in out-of-distribution predictions, especially for emotions less commonly evoked by national anthems. We acknowledge that this may limit the validity of applying the model to this specific musical genre. Sixth, the absence of a baseline for comparison with other genres of music from the same countries limits our ability to determine whether the observed emotional patterns are anthem-specific or reflect broader national musical tendencies. Given the global scope of the study, compiling representative musical corpora for all 176 countries would be unfeasible. We therefore urge caution in interpreting the emotional profiles of anthems as culturally unique without such comparative context. Finally, the extracted musical features may not fully capture all aspects of music that shape emotional perception. Traditional MIR techniques often fall short in representing elements such as subtle articulation, thematic development, gradual structural shifts, and cross-modal associations. While our approach used a “bag-of-frames” representation that averages features across time, we acknowledge that more recent MIR methods incorporating segment-level or temporally sensitive representations may better capture within-song variations relevant to emotional perception and could enhance future modeling efforts.

Apart from methodological limitations, cross-cultural comparisons face several challenges, including ethnocentrism of the researchers. The research team is composed predominantly of individuals who are White and influenced by Western academic traditions. We recognize that our cultural, academic, and socioeconomic backgrounds may shape our perspectives and interpretations, potentially introducing biases into our analysis. Further, with our results, we do not aim to make unwarranted overly simplified claims about specific nations or cultures contributing to stereotypes or prejudice. Especially the complexity of the concept of culture presents difficulties for cultural research, as culture is a multifaceted and nuanced construct with a constantly evolving nature. Accordingly, Hofstede’s theory has received criticism as well, regarding its overgeneralization and limited scope, static nature and Western bias^49^. In particular, the model has been criticized for limitations regarding the original sample of IBM employees underlying the factor analysis, as well as its constricted and outdated assumptions about culture and cultural differences^49^. In contrast, the Inglehart–Welzel cultural map of the world is based on cross-cultural variation in fundamental values placing nations on two axes of secular-rational values versus traditional values, and self-expression values versus survival values^50^. Future studies might therefore consider investigating differences in national anthems in relation to this more dynamic framework. Additionally, a cultural classification of nations might also benefit from alternative frameworks that consider decolonial perspectives. For instance, Aníbal Quijano’s idea of Coloniality of Power explores how the remnants of colonialism continue to shape global hierarchies and power relations, even after formal colonial rule has ended^51^.

Another important consideration regarding Hofstede’s dimensions is that the data were collected during time periods (1967–1973 and 2000-2004^52^) that are significantly removed from those in which most of the analyzed national anthems were composed and therefore reflect very different societal contexts. For example, many South American anthems date back to the early 19th century, well before the major waves of immigration from the Mediterranean and Eastern Europe, which would later have a profound impact on the demographic makeup and cultural identity of these nations.

Furthermore, the history, creation and adoption of national anthems vary greatly between countries, influenced by historical context, political elites, and cultural diversity within nations. Additionally, while this analysis focused on the emotions reflected in an important national symbol, national anthems do not necessarily reflect the population’s current (or former) sentiment and identification with their national anthems. For instance, a marginalized group in Israeli society appears to view the anthem more negatively compared to mainstream Israelis^53^. Further, singing the national anthem has sparked protests in some countries and criticisms related to (forced) patriotism^54^.

### Implications and future directions

Since the aim of this study was to uncover associations using computational modeling, it can serve as a starting point for future research. Based on our computational findings, further studies might now include the viewpoints of specific nations to integrate the voice of the culture and nation itself. For instance, the sentiment toward one’s own national anthem could vary substantially among its own population (ranging from nationalism and pride over indifference to discontent and rage). It might therefore be worthwhile to conduct studies based on self-report data about the sound and emotions directly evoked by the anthem. Further, experimental research could manipulate the musical features of anthems to examine their direct emotional impact. In addition, based on the cultural (and often historical) overlap and similarity within regions of different nations, it might be interesting to focus on the investigation of anthems in specific continents or regions. Future studies might consider grouping countries by cultural-geographical regions or by socioeconomic and political classifications—such as those used by the World Bank^55^—as these alternative frameworks may offer more meaningful bases for interpreting emotional patterns in relation to shared historical, cultural, or structural contexts. In a similar vein, future research could incorporate additional cultural frameworks, such as tightness–looseness and relational mobility, to better capture how social norms and interpersonal dynamics relate to emotional expressions in national anthems.

Furthermore, future research could further explore emotions expressed by national anthems by integrating musical analysis and lyric analysis through advanced computational methods such as latent semantic analysis (LSA), deep learning, and multimodal processing, allowing for deeper insights into the interplay between musical structure and lyrical semantics^56,57^. Cross-cultural perception studies could investigate how individuals from different cultural backgrounds interpret and emotionally respond to different musical anthems, considering factors such as familiarity with the music and sentiment toward the countries. Further, temporal and historical analysis could provide insights into how political, social, and cultural shifts might have influenced the emotional content of national anthems and other politically significant music, examining e.g. how harmonic choices and rhythmic structures relate to musical trends and historical events. In this context, it would also be valuable to examine whether the emotional content of anthems relates to the period in which they were composed, although such analysis was beyond the scope of the present study. Future work could investigate whether changes in musical-emotional characteristics align with political or cultural shifts across historical eras.

Our study also has valuable implications for broader intercultural music research. While national anthems often evoke strong feelings of patriotism and unity, they can also be critiqued for promoting a nationalized worldview overshadowing global interconnectedness, and for legitimizing power and authority over a nation’s people. On the other hand, cultural music in general is deeply embedded in a nation’s heritage, helping to preserve traditions and deepen a sense of community. Since the creation and adoption of national anthems are influenced by a variety of sociological, political and historical factors^3^, folk and traditional music might be more representative of the population itself in a nation. MER could therefore be used as a computational approach to examining and comparing musical differences in folk and traditional music as one possible reflection of a nation.

## Electronic supplementary material

Below is the link to the electronic supplementary material.

Supplementary Material 1

## Data Availability

The recordings of the national anthems were retrieved from https://nationalanthems.info/. Predicted emotional characteristics for each country’s anthem are available at https://osf.io/wz65t/files/osfstorage? view_only=77e7a80975774522879a67ad4b1cdfb4Scripts used for the analysis are available from the first author upon request.
