# Supplementary material for: The emotional geography of National anthems
Source: Sci Rep. 2025 Jul 2;15:23050. doi: 10.1038/s41598-025-08956-6 (PMC12214893; doi:10.1038/s41598-025-08956-6)
Supplement: Supplementary file 1 — Supplementary Material 1 [file 41598_2025_8956_MOESM1_ESM.docx]

**THE EMOTIONAL GEOGRAPHY OF NATIONAL ANTHEMS**

Petri Toiviainen*, Martín Hartmann, & Friederike Koehler

Centre of Excellence in Music, Mind, Body and Brain

Department of Music, Art and Culture Studies

University of Jyväskylä

PO Box 35

40014 University of Jyväskylä

Finland

* corresponding author, email: petri.toiviainen@jyu.fi

# SUPPLEMENTARY MATERIAL

**Supplementary Table 1.** The 176 countries whose national anthems were included in the analysis. Numbers indicate countries for which data on each of Hofstede's scales were available: ^1^PDI, IDV, MAS and UAI (N = 66); ^2^LTO (N = 89); ^3^IVR (N = 89).

^1^Power Distance (PDI), Individualism vs. Collectivism (IDV), Motivation towards Achievement and Success, formerly Masculinity vs. Femininity (MAS), Uncertainty Avoidance (UAI); ^2^Long-Term vs. Short-Term Orientation (LTO), and ^3^Indulgence vs. Restraint (IVR).

| Afghanistan | Dominican Republic^2,3^ | Liechtenstein | Russia^1,2,3^ |
| --- | --- | --- | --- |
| Albania^1,2,3^ | Ecuador^1^ | Lithuania^1,2,3^ | Rwanda^2,3^ |
| Algeria^2,3^ | Egypt^2,3^ | Luxembourg^1,2,3^ | Saint Kitts & Nevis |
| Angola | El Salvador^1,2,3^ | Macedonia^2,3^ | Saudi Arabia^2,3^ |
| Antigua | Eritrea | Madagascar | Senegal |
| Argentina^1,2,3^ | Estonia^1,2,3^ | Malawi | Serbia^1,2,3^ |
| Aruba | Eswatini | Malaysia^1,2,3^ | Seychelles |
| Australia^1,2,3^ | Ethiopia^3^ | Maldives | Sierra Leone |
| Austria^1,2,3^ | Fiji | Mali^2,3^ | Singapore^1,2,3^ |
| Azerbaijan^2,3^ | Finland^1,2,3^ | Malta^1,2,3^ | Slovakia^1,2,3^ |
| Bahamas | France^1,2,3^ | Mauritania | Slovenia^1,2,3^ |
| Bangladesh^1,2,3^ | Gabon | Mauritius | Somalia |
| Barbados | Gambia | Mexico^1,2,3^ | South Korea^1,2,3^ |
| Belarus^2,3^ | Georgia^2,3^ | Micronesia | South Sudan |
| Belgium^1,2,3^ | Germany^1,2,3^ | Moldova^2,3^ | Spain^1,2,3^ |
| Belize | Ghana^2,3^ | Monaco | Sri Lanka |
| Benin | Greece^1,2,3^ | Mongolia | Sudan |
| Bolivia | Guatemala^1^ | Montenegro^2,3^ | Sweden^1,2,3^ |
| Bosnia & Herzegovina^2,3^ | Guinea | Morocco^1,2,3^ | Switzerland^1,2,3^ |
| Botswana | Guinea-Bissau | Mozambique | Syria |
| Brazil^1,2,3^ | Guyana | Myanmar | Tanzania^2,3^ |
| Brunei | Haiti | Namibia | Thailand^1,2,3^ |
| Bulgaria^1,2,3^ | Honduras | Nepal | Timor-Leste |
| Burkina Faso^2,3^ | Hungary^1,2,3^ | Netherlands^1,2,3^ | Togo |
| Cambodia | Iceland^2,3^ | Netherlands Antilles | Trinidad & Tobago^1,2,3^ |
| Cameroon | India^1,2,3^ | New Zealand^1,2,3^ | Tunisia |
| Canada^1,2,3^ | Indonesia^1,2,3^ | Nicaragua | Turkey^1,2,3^ |
| Cape Verde | Iran^1,2,3^ | Niger | Turkmenistan |
| Centr. Afr. Republic | Iraq^2,3^ | Nigeria^2,3^ | Uganda^2,3^ |
| Chad | Ireland^1,2,3^ | Norway^1,2,3^ | UK^1,2,3^ |
| Chile^1,2,3^ | Israel^1,2^ | Oman | Ukraine^2,3^ |
| China^1,2,3^ | Italy^1,2,3^ | Pakistan^1,2,3^ | United Arab Emirates |
| Colombia^1,2,3^ | Jamaica^1^ | Panama^1^ | Uruguay^1,2,3^ |
| Comoros | Japan^1,2,3^ | Papua New Guinea | USA^1,2,3^ |
| Cook Islands | Jordan^2,3^ | Paraguay | Uzbekistan |
| Costa Rica^1^ | Kazakhstan | Peru^1,2,3^ | Vanuatu |
| Cote d'Ivoire | Kenya | Philippines^1,2,3^ | Vatican |
| Croatia^1,2,3^ | Kuwait | Poland^1,2,3^ | Venezuela^1,2,3^ |
| Cuba | Kyrgyzstan^2,3^ | Portugal^1,2,3^ | Vietnam^1,2,3^ |
| Czech^1,2,3^ | Laos | Puerto Rico^2,3^ | Virgin Islands |
| Dem. Rep. of Congo | Latvia^1,2,3^ | Qatar | Western Sahara |
| Denmark^1,2,3^ | Lebanon | Rep. of Congo | Yemen |
| Djibouti | Liberia | Rep. of South Africa^2,3^ | Zambia^2,3^ |
| Dominica | Libya | Romania^1,2,3^ | Zimbabwe^2,3^ |

**Supplementary Table 2.** Predicted emotions for each country's anthem.

| Country | Valence | Energy | Tension | Anger | Fear | Happiness | Sadness | Tenderness |
| --- | --- | --- | --- | --- | --- | --- | --- | --- |
| United Arab Emirates | 4.56 | 5.74 | 3.99 | 2.79 | 1.71 | 4.40 | 0.87 | 1.98 |
| Afghanistan | 4.69 | 4.78 | 3.73 | 2.65 | 1.89 | 2.75 | 2.76 | 2.50 |
| Antigua and Barbuda | 5.28 | 4.40 | 3.17 | 1.59 | 1.25 | 4.66 | 1.40 | 3.61 |
| Albania | 4.95 | 4.78 | 3.54 | 2.40 | 1.42 | 3.97 | 1.52 | 3.04 |
| Netherlands Antilles | 4.46 | 4.43 | 3.94 | 3.18 | 2.19 | 3.52 | 1.51 | 2.59 |
| Angola | 4.74 | 4.89 | 3.78 | 2.59 | 1.49 | 3.17 | 2.05 | 2.58 |
| Argentina | 4.45 | 3.95 | 3.83 | 2.39 | 2.73 | 2.75 | 2.15 | 3.39 |
| Austria | 4.44 | 3.45 | 3.74 | 2.59 | 2.39 | 2.54 | 2.56 | 3.07 |
| Australia | 5.07 | 4.54 | 3.32 | 2.12 | 1.72 | 4.25 | 1.35 | 3.26 |
| Aruba | 5.24 | 3.71 | 3.16 | 1.74 | 2.03 | 3.20 | 2.57 | 3.78 |
| Azerbaijan | 4.31 | 4.73 | 4.18 | 3.04 | 2.11 | 2.09 | 2.91 | 2.51 |
| Bosnia and Herzegovina | 4.73 | 3.31 | 3.66 | 2.66 | 2.83 | 2.37 | 2.42 | 3.78 |
| Barbados | 4.57 | 5.04 | 3.73 | 2.97 | 1.64 | 4.32 | 1.05 | 2.68 |
| Bangladesh | 4.53 | 4.45 | 4.00 | 2.26 | 2.16 | 2.48 | 2.37 | 2.24 |
| Belgium | 4.47 | 4.84 | 4.02 | 3.32 | 2.03 | 3.43 | 1.67 | 2.58 |
| Burkina Faso | 4.80 | 4.82 | 3.71 | 2.61 | 1.69 | 3.72 | 1.62 | 3.04 |
| Bulgaria | 4.23 | 4.60 | 4.33 | 3.33 | 2.14 | 2.33 | 2.80 | 2.35 |
| Benin | 4.75 | 5.24 | 3.76 | 2.64 | 1.65 | 4.15 | 1.01 | 2.64 |
| Brunei Darussalam | 4.15 | 4.58 | 4.34 | 3.56 | 2.29 | 3.10 | 1.71 | 2.41 |
| Bolivia | 4.33 | 5.35 | 4.06 | 3.04 | 1.95 | 4.02 | 0.74 | 2.14 |
| Brazil | 4.01 | 5.46 | 4.39 | 2.72 | 2.46 | 3.40 | 1.29 | 2.29 |
| Bahamas | 4.15 | 5.15 | 4.18 | 3.53 | 2.36 | 3.89 | 0.81 | 2.25 |
| Botswana | 5.08 | 4.70 | 3.32 | 2.45 | 1.51 | 4.29 | 1.17 | 3.04 |
| Belarus | 5.15 | 5.06 | 3.55 | 2.97 | 1.29 | 4.12 | 1.17 | 3.17 |
| Belize | 4.67 | 4.86 | 3.64 | 2.96 | 1.70 | 3.85 | 1.43 | 2.74 |
| Canada | 4.58 | 4.74 | 3.98 | 2.98 | 1.85 | 3.35 | 1.74 | 2.77 |
| Central African Republic | 3.91 | 5.27 | 4.52 | 3.27 | 2.45 | 3.33 | 1.26 | 2.23 |
| Congo | 4.97 | 4.69 | 3.53 | 2.74 | 1.48 | 3.67 | 1.77 | 3.15 |
| Switzerland | 4.80 | 3.96 | 3.71 | 2.88 | 2.60 | 3.63 | 1.52 | 3.50 |
| Cote d'Ivoire | 4.47 | 5.41 | 4.01 | 2.73 | 2.24 | 3.94 | 0.94 | 2.61 |
| Cook Islands | 5.04 | 5.30 | 3.54 | 2.91 | 1.25 | 4.57 | 0.46 | 3.20 |
| Chile | 3.93 | 5.81 | 4.64 | 3.57 | 2.80 | 3.87 | 0.44 | 1.90 |
| Cameroon | 4.58 | 5.24 | 3.81 | 2.87 | 1.80 | 4.16 | 0.85 | 2.51 |
| China | 5.38 | 5.82 | 3.41 | 2.12 | 0.77 | 5.47 | 0.23 | 3.00 |
| Colombia | 4.53 | 4.81 | 3.83 | 2.44 | 1.84 | 3.37 | 1.87 | 2.86 |
| Costa Rica | 3.87 | 5.28 | 4.61 | 3.32 | 2.83 | 3.45 | 0.75 | 2.10 |
| Cuba | 4.47 | 5.64 | 4.19 | 2.81 | 2.14 | 4.09 | 0.70 | 2.20 |
| Cape Verde | 5.06 | 5.07 | 3.60 | 3.01 | 1.52 | 4.20 | 0.85 | 3.12 |
| Czech Republic | 5.28 | 3.95 | 3.21 | 1.57 | 2.00 | 4.08 | 1.46 | 4.05 |
| Germany | 4.80 | 4.41 | 3.75 | 2.86 | 2.08 | 3.67 | 1.51 | 2.96 |
| Djibouti | 4.71 | 5.70 | 3.90 | 2.93 | 1.52 | 4.23 | 0.82 | 2.48 |
| Denmark | 4.48 | 4.11 | 3.82 | 2.71 | 2.44 | 3.23 | 2.02 | 3.15 |
| Dominica | 5.00 | 5.29 | 3.59 | 3.32 | 1.27 | 5.03 | 0.26 | 3.10 |
| Dominican Republic | 4.72 | 5.03 | 3.72 | 2.36 | 1.75 | 4.03 | 1.21 | 3.03 |
| Algeria | 4.36 | 5.25 | 4.07 | 3.20 | 1.98 | 3.51 | 1.24 | 2.19 |
| Ecuador | 4.23 | 5.08 | 4.11 | 3.01 | 2.41 | 3.38 | 1.41 | 2.55 |
| Estonia | 5.02 | 5.39 | 3.45 | 2.69 | 0.77 | 4.88 | 0.69 | 2.76 |
| Egypt | 4.86 | 4.63 | 3.41 | 2.33 | 1.42 | 3.66 | 1.85 | 3.31 |
| Western Sahara | 5.64 | 5.02 | 2.60 | 1.67 | 0.82 | 5.64 | 0.46 | 3.83 |
| Eritrea | 4.98 | 4.82 | 3.38 | 2.52 | 1.67 | 3.78 | 1.32 | 2.87 |
| Spain | 4.93 | 5.18 | 3.77 | 2.67 | 1.14 | 3.98 | 1.21 | 2.74 |
| Ethiopia | 4.97 | 5.12 | 3.54 | 2.76 | 1.45 | 3.59 | 1.70 | 2.86 |
| Finland | 4.66 | 5.00 | 3.70 | 2.60 | 1.62 | 4.04 | 1.23 | 2.97 |
| Fiji | 5.20 | 4.88 | 3.50 | 2.44 | 1.47 | 4.99 | 0.55 | 3.34 |
| Micronesia, Federated States of | 4.92 | 4.27 | 3.62 | 2.66 | 1.83 | 3.95 | 1.25 | 3.30 |
| France | 4.44 | 5.40 | 3.95 | 3.03 | 1.68 | 4.18 | 0.69 | 2.12 |
| Gabon | 4.16 | 5.04 | 4.24 | 3.10 | 2.64 | 3.37 | 1.22 | 2.34 |
| United Kingdom | 4.33 | 3.21 | 3.99 | 2.79 | 3.22 | 2.11 | 2.73 | 2.84 |
| Georgia | 4.91 | 4.68 | 3.61 | 2.37 | 1.54 | 3.91 | 1.43 | 3.01 |
| Ghana | 4.18 | 5.41 | 4.38 | 3.28 | 2.25 | 3.80 | 0.78 | 2.39 |
| Gambia | 4.82 | 4.15 | 3.40 | 2.53 | 1.71 | 3.81 | 1.83 | 3.55 |
| Guinea | 4.94 | 4.50 | 3.37 | 2.36 | 1.34 | 3.95 | 1.58 | 3.30 |
| Greece | 4.28 | 5.21 | 4.10 | 2.45 | 1.90 | 3.37 | 1.68 | 2.61 |
| Guatemala | 4.52 | 4.44 | 3.91 | 2.19 | 2.33 | 3.34 | 1.79 | 3.23 |
| Guinea-Bissau | 4.85 | 5.34 | 3.91 | 3.15 | 1.71 | 4.51 | 0.40 | 2.59 |
| Guyana | 4.91 | 4.06 | 3.31 | 1.99 | 1.86 | 3.24 | 2.56 | 3.45 |
| Honduras | 4.48 | 4.84 | 4.00 | 2.77 | 2.07 | 3.37 | 1.48 | 2.81 |
| Croatia | 4.83 | 4.17 | 3.59 | 2.57 | 1.99 | 3.78 | 1.57 | 3.28 |
| Haiti | 3.88 | 5.49 | 4.61 | 3.26 | 2.32 | 3.28 | 1.54 | 1.66 |
| Hungary | 4.83 | 3.28 | 3.41 | 2.11 | 2.54 | 2.62 | 2.78 | 4.21 |
| Indonesia | 4.49 | 5.35 | 4.12 | 3.20 | 2.30 | 4.21 | 0.51 | 2.38 |
| Ireland | 4.33 | 5.55 | 4.18 | 3.29 | 2.02 | 3.99 | 0.80 | 2.22 |
| Israel | 4.17 | 3.36 | 4.13 | 2.89 | 3.20 | 1.24 | 3.77 | 2.81 |
| India | 5.06 | 4.82 | 3.43 | 2.65 | 1.10 | 3.04 | 2.60 | 3.10 |
| Iraq | 4.61 | 4.40 | 3.75 | 2.31 | 2.20 | 2.80 | 2.30 | 3.08 |
| Iran Islamic Republic of | 4.84 | 4.52 | 3.81 | 2.64 | 2.13 | 3.95 | 1.07 | 2.81 |
| Iceland | 4.90 | 4.25 | 3.52 | 2.45 | 2.06 | 3.62 | 1.76 | 3.40 |
| Italy | 4.59 | 5.07 | 3.84 | 2.70 | 1.95 | 3.84 | 1.20 | 2.83 |
| Jamaica | 4.04 | 3.51 | 4.23 | 3.30 | 3.29 | 1.97 | 2.64 | 2.99 |
| Jordan | 4.05 | 4.67 | 4.39 | 3.60 | 2.38 | 2.68 | 1.86 | 2.48 |
| Japan | 5.67 | 2.75 | 2.43 | 1.07 | 1.23 | 2.52 | 3.95 | 4.59 |
| Kenya | 5.55 | 4.42 | 2.87 | 1.90 | 0.70 | 4.00 | 2.24 | 3.60 |
| Kyrgyzstan | 5.22 | 4.04 | 3.30 | 2.13 | 1.87 | 3.45 | 2.26 | 3.56 |
| Cambodia | 5.60 | 3.27 | 2.66 | 1.04 | 1.45 | 3.09 | 3.33 | 4.49 |
| Comoros | 4.66 | 5.59 | 3.99 | 3.07 | 1.89 | 4.20 | 0.81 | 2.63 |
| Saint Kitts and Nevis | 4.68 | 5.83 | 3.98 | 3.13 | 1.75 | 4.52 | 0.46 | 2.31 |
| Korea, Republic of | 4.99 | 4.54 | 3.52 | 2.49 | 1.77 | 3.92 | 1.41 | 3.25 |
| Kuwait | 4.29 | 5.84 | 4.24 | 3.35 | 2.04 | 4.18 | 0.69 | 1.48 |
| Kazakhstan | 4.45 | 5.36 | 4.08 | 3.11 | 1.67 | 2.88 | 2.27 | 2.19 |
| Lao People's Democratic Republic | 4.64 | 5.24 | 3.76 | 2.64 | 1.63 | 3.82 | 1.43 | 2.86 |
| Lebanon | 4.51 | 5.65 | 3.99 | 3.41 | 1.96 | 4.66 | 0.17 | 2.53 |
| Liechtenstein | 4.25 | 3.04 | 3.81 | 2.41 | 3.22 | 1.50 | 3.38 | 2.88 |
| Sri Lanka | 4.76 | 4.46 | 3.74 | 2.63 | 2.24 | 3.37 | 1.88 | 3.08 |
| Liberia | 4.77 | 5.34 | 3.65 | 3.05 | 1.62 | 4.61 | 0.48 | 2.99 |
| Lithuania | 4.38 | 4.94 | 4.03 | 2.96 | 1.88 | 3.80 | 1.06 | 2.62 |
| Luxembourg | 4.80 | 2.67 | 3.39 | 2.37 | 2.94 | 2.56 | 2.61 | 3.91 |
| Latvia | 4.69 | 4.21 | 3.53 | 2.56 | 1.75 | 3.41 | 1.79 | 3.09 |
| Libyan Arab Jamahiriya | 4.33 | 5.39 | 3.93 | 2.76 | 1.94 | 3.85 | 0.96 | 1.99 |
| Morocco | 4.56 | 4.78 | 3.85 | 2.55 | 1.81 | 2.69 | 2.83 | 2.39 |
| Monaco | 4.35 | 5.47 | 4.09 | 2.98 | 2.24 | 3.85 | 0.90 | 2.21 |
| Republic of Moldova | 4.31 | 4.26 | 4.25 | 3.06 | 2.69 | 2.57 | 2.09 | 2.52 |
| Montenegro | 4.39 | 5.21 | 4.20 | 3.25 | 1.83 | 3.23 | 1.45 | 2.24 |
| Madagascar | 4.44 | 5.03 | 4.05 | 3.07 | 2.10 | 3.63 | 1.17 | 2.60 |
| The former Yugoslav Republic of Macedonia | 4.76 | 4.75 | 3.88 | 2.74 | 1.80 | 3.28 | 1.73 | 2.75 |
| Mali | 4.50 | 4.34 | 3.75 | 2.94 | 2.01 | 2.76 | 2.23 | 2.98 |
| Burma | 4.68 | 5.01 | 3.84 | 3.15 | 1.70 | 4.18 | 0.95 | 2.69 |
| Mongolia | 4.51 | 4.44 | 3.84 | 2.61 | 1.97 | 2.74 | 2.14 | 2.84 |
| Mauritania | 4.33 | 5.53 | 3.99 | 2.45 | 1.81 | 3.73 | 1.44 | 2.20 |
| Malta | 4.97 | 3.63 | 3.46 | 2.37 | 2.08 | 2.76 | 2.72 | 3.36 |
| Mauritius | 4.07 | 4.53 | 4.32 | 3.00 | 2.65 | 2.90 | 1.92 | 2.21 |
| Maldives | 4.98 | 5.02 | 3.67 | 2.37 | 1.55 | 3.95 | 1.25 | 2.75 |
| Malawi | 5.30 | 4.70 | 3.27 | 2.41 | 1.09 | 4.70 | 0.90 | 3.16 |
| Mexico | 4.45 | 5.08 | 3.99 | 2.81 | 1.92 | 3.74 | 1.28 | 2.66 |
| Malaysia | 4.29 | 4.97 | 4.17 | 3.04 | 2.43 | 3.65 | 1.14 | 2.13 |
| Mozambique | 4.47 | 5.39 | 4.10 | 3.34 | 2.00 | 3.63 | 1.07 | 2.49 |
| Namibia | 5.48 | 3.82 | 3.15 | 2.41 | 1.82 | 3.84 | 1.78 | 4.16 |
| Niger | 5.15 | 4.88 | 3.07 | 1.74 | 0.61 | 3.58 | 2.39 | 3.46 |
| Nigeria | 3.71 | 5.66 | 4.96 | 3.89 | 2.99 | 3.06 | 1.03 | 1.22 |
| Nicaragua | 3.67 | 5.12 | 4.91 | 3.51 | 3.15 | 2.92 | 1.01 | 1.84 |
| Netherlands | 5.64 | 3.53 | 2.64 | 1.18 | 1.48 | 4.08 | 2.02 | 4.48 |
| Norway | 4.91 | 4.38 | 3.55 | 2.75 | 1.82 | 3.16 | 2.63 | 3.11 |
| Nepal | 4.95 | 5.04 | 3.57 | 2.50 | 1.44 | 3.46 | 1.84 | 2.64 |
| New Zealand | 4.69 | 4.54 | 3.85 | 2.85 | 2.28 | 4.10 | 1.09 | 2.85 |
| Oman | 5.34 | 4.70 | 2.94 | 2.07 | 0.77 | 4.91 | 0.96 | 3.93 |
| Panama | 4.34 | 5.39 | 4.22 | 3.67 | 2.21 | 3.99 | 0.61 | 2.40 |
| Peru | 4.25 | 4.56 | 4.18 | 2.86 | 2.64 | 3.09 | 1.68 | 2.63 |
| Papua New Guinea | 4.60 | 5.04 | 3.69 | 2.64 | 1.62 | 4.19 | 0.93 | 2.52 |
| Philippines | 4.83 | 5.41 | 3.67 | 2.69 | 1.46 | 4.80 | 0.34 | 3.12 |
| Pakistan | 4.09 | 4.88 | 4.41 | 3.29 | 2.39 | 2.79 | 1.84 | 2.16 |
| Poland | 4.92 | 5.42 | 3.57 | 2.57 | 1.28 | 4.71 | 0.71 | 2.90 |
| Puerto Rico | 4.48 | 3.84 | 3.83 | 2.14 | 2.65 | 2.21 | 2.80 | 3.28 |
| Portugal | 4.89 | 4.98 | 3.50 | 2.76 | 1.50 | 4.48 | 0.98 | 3.14 |
| Paraguay | 4.20 | 4.52 | 4.12 | 2.55 | 2.66 | 2.94 | 1.80 | 2.70 |
| Qatar | 3.14 | 5.07 | 5.29 | 3.91 | 3.63 | 2.12 | 1.49 | 1.27 |
| Romania | 4.81 | 5.55 | 3.69 | 2.91 | 1.26 | 4.14 | 1.34 | 2.30 |
| Serbia | 4.55 | 5.04 | 3.98 | 2.97 | 1.75 | 3.49 | 1.69 | 2.46 |
| Russia | 4.47 | 4.91 | 4.13 | 2.90 | 2.13 | 3.07 | 1.96 | 2.90 |
| Rwanda | 4.69 | 5.81 | 4.06 | 3.43 | 1.58 | 4.67 | 0.23 | 2.48 |
| Saudi Arabia | 4.48 | 5.80 | 4.11 | 2.86 | 1.68 | 4.49 | 0.56 | 2.23 |
| Seychelles | 4.89 | 4.57 | 3.70 | 2.74 | 1.80 | 4.10 | 0.94 | 3.01 |
| Sudan | 3.60 | 5.25 | 4.92 | 3.91 | 3.08 | 3.03 | 0.68 | 1.64 |
| Sweden | 5.30 | 3.92 | 3.27 | 2.36 | 2.03 | 3.62 | 1.56 | 3.83 |
| Singapore | 4.89 | 5.27 | 3.70 | 2.94 | 1.51 | 4.67 | 0.77 | 2.69 |
| Slovenia | 5.08 | 4.46 | 3.30 | 1.95 | 1.48 | 3.73 | 1.95 | 3.18 |
| Slovakia | 4.97 | 5.65 | 3.76 | 2.47 | 1.19 | 4.39 | 1.28 | 2.45 |
| Sierra Leone | 4.91 | 5.32 | 3.68 | 2.29 | 1.05 | 4.29 | 1.23 | 2.96 |
| Senegal | 4.31 | 5.04 | 4.24 | 3.39 | 2.03 | 3.32 | 1.66 | 2.07 |
| Somalia | 5.01 | 3.95 | 3.18 | 1.73 | 1.48 | 3.12 | 2.43 | 3.42 |
| South Sudan | 4.47 | 4.81 | 4.05 | 2.98 | 2.18 | 3.19 | 1.51 | 2.21 |
| El Salvador | 3.78 | 5.38 | 4.74 | 3.68 | 2.91 | 3.13 | 0.94 | 1.96 |
| Syrian Arab Republic | 4.45 | 4.79 | 3.95 | 2.99 | 1.82 | 2.77 | 2.45 | 2.79 |
| Swaziland | 4.78 | 4.94 | 3.59 | 2.07 | 1.30 | 3.95 | 1.83 | 2.83 |
| Chad | 4.45 | 5.58 | 3.95 | 3.04 | 1.74 | 3.66 | 1.00 | 2.17 |
| Togo | 4.53 | 5.12 | 4.01 | 3.09 | 2.05 | 3.51 | 1.30 | 2.29 |
| Thailand | 4.91 | 4.60 | 3.55 | 2.74 | 1.67 | 3.96 | 1.32 | 2.95 |
| Timor-Leste | 5.09 | 4.39 | 3.45 | 2.62 | 1.75 | 3.73 | 1.39 | 3.38 |
| Turkmenistan | 4.97 | 4.96 | 3.62 | 2.03 | 1.51 | 4.11 | 1.14 | 2.87 |
| Tunisia | 4.60 | 5.35 | 3.82 | 3.14 | 1.55 | 3.63 | 1.64 | 2.20 |
| Turkey | 4.31 | 5.17 | 4.05 | 2.78 | 1.69 | 3.13 | 1.89 | 2.34 |
| Trinidad and Tobago | 4.67 | 4.26 | 3.74 | 2.78 | 1.97 | 3.02 | 2.38 | 3.15 |
| United Republic of Tanzania | 5.18 | 4.65 | 3.28 | 2.03 | 1.69 | 4.58 | 1.14 | 3.46 |
| Ukraine | 4.97 | 5.21 | 3.61 | 2.53 | 1.12 | 4.05 | 1.61 | 2.87 |
| Uganda | 5.05 | 3.30 | 3.22 | 2.41 | 1.99 | 2.73 | 2.53 | 4.26 |
| United States | 4.29 | 4.66 | 4.07 | 3.04 | 2.35 | 3.02 | 1.80 | 2.65 |
| Uruguay | 4.29 | 4.87 | 4.18 | 2.84 | 2.36 | 3.26 | 1.57 | 2.55 |
| Uzbekistan | 4.38 | 4.93 | 4.16 | 2.87 | 2.28 | 2.62 | 2.31 | 2.56 |
| Holy See Vatican City | 4.82 | 5.14 | 3.61 | 2.86 | 1.39 | 4.37 | 0.73 | 3.31 |
| Venezuela | 4.57 | 5.67 | 4.14 | 3.06 | 1.65 | 4.16 | 0.75 | 2.50 |
| United States Virgin Islands | 3.56 | 5.40 | 4.95 | 3.47 | 3.06 | 2.56 | 1.55 | 1.42 |
| Viet Nam | 3.80 | 4.40 | 4.63 | 3.29 | 3.20 | 2.15 | 2.26 | 2.03 |
| Vanuatu | 5.43 | 5.00 | 3.12 | 2.13 | 0.90 | 4.78 | 1.41 | 3.82 |
| Yemen | 4.92 | 4.71 | 3.52 | 2.18 | 1.23 | 3.23 | 2.14 | 2.58 |
| South Africa | 5.17 | 4.60 | 3.48 | 2.47 | 1.55 | 4.17 | 1.39 | 3.35 |
| Zambia | 5.19 | 4.50 | 3.30 | 2.04 | 1.92 | 4.39 | 1.32 | 3.46 |
| Democratic Republic of the Congo | 5.03 | 4.70 | 3.54 | 2.59 | 1.71 | 4.32 | 1.08 | 3.23 |
| Zimbabwe | 5.29 | 4.51 | 3.20 | 1.76 | 0.81 | 5.00 | 1.08 | 3.27 |


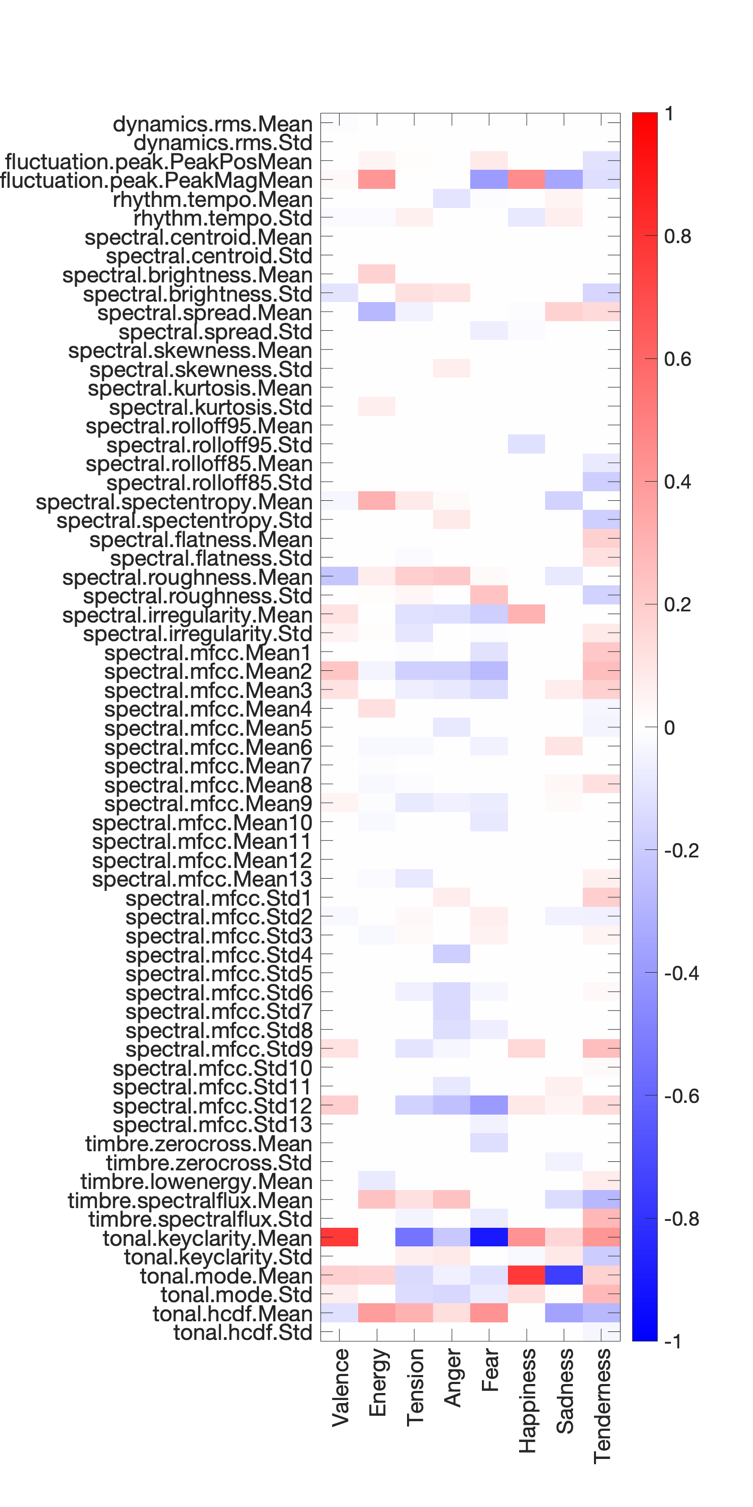


**Supplementary Figure 1.** Normalized beta coefficients obtained with LASSO regression for each of the extracted musical features and each of the eight emotion characteristics. As can be seen, musical features related to tonality and rhythmic fluctuation contribute most to the prediction of emotions. For explanation of the musical feature names, please refer to the MIR Toolbox manual (Lartillot & Toiviainen 2007).
